# Increased Depth of Cellular Imaging in the Intact Lung Using Far-Red and Near-Infrared Fluorescent Probes

**DOI:** 10.1155/IJBI/2006/37470

**Published:** 2006-03-08

**Authors:** Abu-Bakr Al-Mehdi, Mita Patel, Abu Haroon, Darla Reed, B. Ohlsson-Wilhelm, K. Muirhead, Brian D. Gray

**Affiliations:** ^1^ Center for Lung Biology and Department of Pharmacology, College of Medicine, University of South Alabama, 307 N. University Boulevand, MSB 3370, Mobile, AL 36688, USA; ^2^ SciGro, Inc., Malvern, PA 19355, USA; ^3^ PTI Research, Inc., Exton, PA 19341, USA

## Abstract

Scattering of shorter-wavelength visible light limits the fluorescence imaging depth of thick specimens such as whole organs. In this study, we report the use of four newly synthesized near-infrared and far-red fluorescence probes (excitation/emission, in nm: 644/670; 683/707; 786/814; 824/834) to image tumor cells in the subpleural vasculature of the intact rat lungs. Transpelural imaging of tumor cells labeled with long-wavelength probes and expressing green fluorescent protein (GFP; excitation/emission 488/507 nm) was done in the intact rat lung after perfusate administration or intravenous injection. Our results show that the average optimum imaging depth for the long-wavelength probes is higher (27.8 ± 0.7 μm) than for GFP (20 ± 0.5 μm; p = 0.008; n = 50), corresponding to a 40% increase in the volume of tissue accessible for high-resolution imaging. The maximum depth of cell visualization was significantly improved with the novel dyes (36.4 ± 1 μm from the pleural surface) compared with GFP (30.1 ± 0.5 μm; p = 0.01; n = 50). Stable binding of the long-wavelength vital dyes to the plasma membrane also permitted in vivo tracking of injected tumor cells in the pulmonary vasculature. These probes offer a significant improvement in the imaging quality of in situ biological processes in the deeper regions of intact lungs.


Hindawi Publishing Corporation
International Journal of Biomedical Imaging
Volume 2006, Article ID 37470, Pages 1–7
DOI 10.1155/IJBI/2006/37470

### Increased Depth of Cellular Imaging in the Intact Lung Using Far-Red and Near-Infrared Fluorescent Probes

**Abu-Bakr Al-Mehdi,[1] Mita Patel,[1] Abu Haroon,[1] Darla Reed,[1] B. Ohlsson-Wilhelm,[2] K. Muirhead,[2] and Brian D. Gray[3]**

[1] *Center for Lung Biology and Department of Pharmacology, College of Medicine, University of South Alabama, 307 N. University Boulevand, MSB 3370, Mobile, AL 36688, USA*
[2] *SciGro, Inc., Malvern, PA 19355, USA*
[3] *PTI Research, Inc., Exton, PA 19341, USA*

Received 19 July 2005; Accepted 18 October 2005

Recommended for Publication by Yue Wang

Scattering of shorter-wavelength visible light limits the fluorescence imaging depth of thick specimens such as whole organs. In this study, we report the use of four newly synthesized near-infrared and far-red fluorescence probes (excitation/emission, in nm: 644/670; 683/707; 786/814; 824/834) to image tumor cells in the subpleural vasculature of the intact rat lungs. Transpelural imaging of tumor cells labeled with long-wavelength probes and expressing green fluorescent protein (GFP; excitation/emission 488/507 nm) was done in the intact rat lung after perfusate administration or intravenous injection. Our results show that the average optimum imaging depth for the long-wavelength probes is higher ($27.8 \pm 0.7\,\mu$m) than for GFP ($20 \pm 0.5\,\mu$m; $p = 0.008$; $n = 50$), corresponding to a 40% increase in the volume of tissue accessible for high-resolution imaging. The maximum depth of cell visualization was significantly improved with the novel dyes ($36.4 \pm 1\,\mu$m from the pleural surface) compared with GFP ($30.1 \pm 0.5\,\mu$m; $p = 0.01$; $n = 50$). Stable binding of the long-wavelength vital dyes to the plasma membrane also permitted in vivo tracking of injected tumor cells in the pulmonary vasculature. These probes offer a significant improvement in the imaging quality of in situ biological processes in the deeper regions of intact lungs.

Copyright © 2006 Abu-Bakr Al-Mehdi et al. This is an open access article distributed under the Creative Commons Attribution License, which permits unrestricted use, distribution, and reproduction in any medium, provided the original work is properly cited.

### 1. INTRODUCTION

Physiologists understood early that the study of life is better in the living than in fixed cells, tissues, or organs. The ultimate question is not what happens in the test tube or in a frozen moment of time, but what happens kinetically in live cells in situ. It is now evident that fluorescence imaging techniques of live cells and organs have been and will continue to be of major help in answering this question.

Using an experimental metastasis model that involves intravenous injection of tumor cells in animals, we study the interaction of tumor cells with the endothelial cells in the target organ such as the lung and monitor their fate within and outside the blood vessels. Green fluorescence protein and fluorescence probes emitting in the 500–600 nm range have been used to label and detect the tumor cells in the pulmonary circulation in situ [1–3]. With probes in this wavelength range, there is substantial tissue scattering of both exciting light and emitted fluorescence. It was demonstrated that long-wavelength (> 600 nm) probes give the best signal levels in mammalian tissue due to lower absorption in tissue [4]. Scattering in tissues may result from photon interactions with sub-wavelength size structures, such as individual molecules (Raleigh's scattering), or from interaction with supra-wavelength size structures, such as cells, organelles, and cytoskeleton (Mie scattering) [5, 6]. Raleigh scattering is inversely proportional to the fourth power of wavelength; therefore, scattering at 480 nm excitation and 510 nm emission for GFP is 6.97 and 6.36 times more than at 780 nm excitation and 810 nm emission, respectively. Maximum usable imaging depth in the intact lung is less than 30 $\mu$m with a ×60 objective and GFP as a fluorophore due to significant limitation of the penetration depth by scattering. The purpose of these studies was to use far-red and near-infrared fluorescence probes to enhance penetration and increase usable imaging depth in the intact lung. Although hardware for

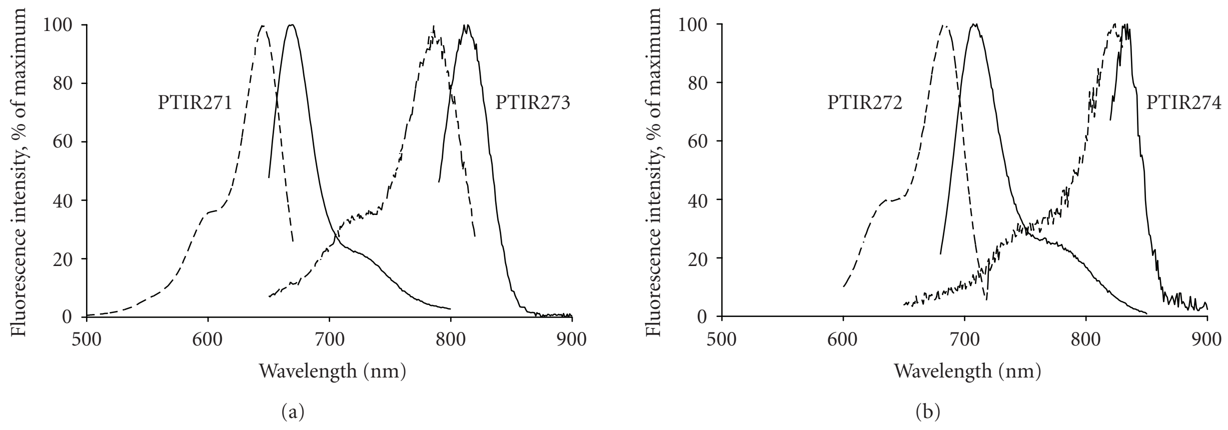

Figure 1: Normalized excitation (dashed lines) and emission (solid lines) spectra of novel far-red and near-IR fluorescence probes. Spectra of 250 nM dye in ethanol were acquired with a Photon Technology International spectrofluorometer (R928 PMT; slit width 4 nm) and normalized-to-peak intensity. Peak excitation and emission wavelengths (nm) were PTIR271–644/670; PTIR272–683/707; PTIR273–786/814; PTIR274–824/834. Small Stokes' shift and low fluorescence intensity made it difficult to get a complete excitation scan of PTIR274.

Table 1: Characteristics of the far-red and near-infrared fluorescence probes and corresponding Chroma Technologies filter sets for microscopy. Bandpass filter sets Cy5 (for PTIR271) and 41020 (for eGFP) are off-the-shelf filters from Chroma Technologies. Sets for PTIR272 to PTIR274 were custom manufactured by Chroma. Their names indicate peak excitation/emission wavelengths. FWHM indicates full width of transmission band at half intensity of maximum transmission.

| Probe | Peak excitation/ emission (nm) | Filter set name | Excitation filter Peak transmission/ FWHM (nm) | Dichroic filter long pass (nm) | Emission filter Peak transmission/ FWHM (nm) |
|---|---|---|---|---|---|
| PTIR271 | 644/670 | Cy5 | D640/20 | 660DCLP | D680/30 |
| PTIR272 | 683/707 | 680/715 | HQ680/20 | Q695LP | HQ715/30 |
| PTIR273 | 786/814 | 780/820 | HQ780/20 | Q800LP | HQ820/30 |
| PTIR274 | 824/834 | 800/840 | HQ800/20 | Q820LP | HQ840/30 |
| eGFP | 488/507 | 41020 | HQ480/20 | Q495LP | HQ510/20 |

imaging in the near-IR and far-red wavelengths is available off-the-shelf or is easily made to order, there has been limited use of any such fluorescent probe in research use for assessing cellular function in an intact organ [7–10]. We have evaluated four newly developed fluorescent probes of near-IR and far-red range to test their utility for imaging tumor cells at greater depths in the intact rat lung. Our results indicate that, compared with GFP, these dyes offer the advantage of high-resolution imaging of tumor cells at depths greater than 30 $\mu$m from the pleural surface of the intact lung. These vital dyes also appear to be suitable for in vivo tracking of injected tumor cells in the pulmonary vasculature because of their stable binding to the plasma membrane.

### 2. MATERIALS AND METHODS

### *2.1. Fluorescence probes and filter sets*

The vital probes PTIR271, PTIR272, PTIR273, and PTIR274 are membrane-intercalating dyes developed at PTI Research, Inc. (Exton, Pa). Like the visible membrane dyes PKH26 and PKH67, the PTIR dyes label cells by partitioning into lipid regions of cell membranes [11, 12]. However, the PTIR dyes are both excited and fluoresce in the far-red to near-infrared region of the spectrum (Figure 1). Spectra were all run on the HORIBA fluoromax instrument and at settings where there was not significant light-scattering artifact for PTIR272 (spectra currently in this figure were taken on the PTI instrument). Based on these spectra, high-quality bandpass interference filters for use in a Nikon TE2000 inverted widefield epifluorescence microscope were custom made for the dyes PTIR272-274 by Chroma Technologies (Rockingham, Vt) and are described in (Table 1). For PTIR271, the Cy5 filter set was readily usable.

### *2.2. Cells and labeling*

A GFP-expressing murine breast cancer cell line was used to compare the PTIR dyes with the GFP fluorescence for tumor cell localization, depth estimation, and image quality (signal-to-noise ratio). 4T1-GFP cells were trypsinized from T75 flasks, pelleted and washed with Hanks' buffer, and then labeled with PTIR dyes at 5 $\mu$M final concentration in Diluent C (Sigma -Aldrich Chemical Co., St. Louis, Mo) for

5 minutes, after which staining was stopped by addition of serum. The cells were washed again with Hanks' buffer before being administered in the isolated lung or injected intravenously in animals. To assess the effect of the PTIR dyes on viability, labeling was performed in 35 mm Petri dishes. After washing with Hanks' solution, viability was assessed by exclusion of the fluorescent probe propidium iodide, 10 $\mu$M, after 30-minute incubation. The cells did not exhibit signs of loss of viability at 1 or 5 $\mu$M of PTIR dyes (one nonviable cell per about 300 cells). Five $\mu$M concentration was not only nontoxic for these probes, but the resultant fluorescence intensity also allowed high-quality imaging of the cells in the pulmonary vessels of the intact lung, yielding S/N of greater than 10. Signal-to-noise ratio, a measure of the inherent "noisiness" of the region of interest in the picture, was calculated by dividing the background-subtracted average intensity value in the region of interest containing fluorescent cells by the standard deviation of mean pixel intensity for the same region, using the image analysis functions of MetaMorph.

### 2.3. Fluorescence imaging of 4T1-GFP cancer cells in subpleural microvessels in situ in the intact rat lung

An established intact organ microscopy method was utilized to observe and image subpleural pulmonary vessels in situ in the isolated, ventilated, blood-free lungs in real time using an epifluorescence microscope [1, 13]. In brief, for lung isolation, the animal was anesthetized with intraperitoneal injection of 60 mg/kg body weight sodium pentobarbital. A tracheostomy was performed and artificial ventilation with 95% air +5% $CO_2$ was started through a cannula. The abdomen was opened and the animal was exsanguinated by transection of major abdominal vessels. A cannula was inserted into the main pulmonary artery via a puncture in the right ventricle and another was inserted into the left atrium. The lung was cleared of blood by gravity perfusion via the pulmonary artery with an artificial medium (Hanks' solution with 5% dextran and 10 mM glucose at pH 7.4). The flow-through perfusate left the lung via the left atrial cannula. Once the lung became visibly cleared of blood, the heart-lung preparation was dissected en bloc and was placed in a specially designed Plexiglas chamber with ports for the tracheal, pulmonary, and left atrial cannulae. The cardiovascular ports were connected to a peristaltic pump that recirculated 40 ml of perfusate through the pulmonary vascular bed. The lung was suspended sideways over a coverslip window at the bottom of the chamber with the posterior surface of the lung gently touching the coverslip.

The subpleural vasculature of the lungs was directly visualized at high magnification (×600) by epifluorescence microscopy. For imaging the morphological and functional dynamics of PTIR probe-labeled 4T1-GFP cancer cells in the subpleural pulmonary circulation, we used a high-resolution digital fluorescence video microscopy system consisting of a Nikon TE2000 inverted fluorescence microscope, ×60 water immersion (NA is 1.2) and ×60 oil immersion (NA is 1.4) objectives, automated 10-position filter wheels for both excitation and emission (Sutter Instruments, Lambda 10-2), automated dichroic filter cube changer (Nikon), $xy$-axis automated stage (Prior Scientific, Inc.), $z$-axis motor (Prior), a high-resolution, Far-Red and near-IR-sensitive 12-bit C4742-95-12ERG IEEE 1394 digital CCD camera with acquisition rate of 18 frames/s with 2 × 2 binning (Hamamatsu Inc.), and MetaMorph image acquisition, processing and analysis software with 3D reconstruction, and point-spread-function (PSF) -based deconvolution capabilities (Universal Imaging Corp., Downingtown, Pa). For 3D reconstruction, images of the same area were acquired along 40 $\mu$m of $z$-axis at 0.5 $\mu$m intervals (optical slicing) over 40 seconds. However, since this was a widefield fluorescence microscope system, there was some out-of-focus light at each of the planes of acquisition. To eliminate the out-of-focus light, standard 0.1 and 0.5 $\mu$m fluorescent beads were used as point sources of light and $z$-axis image stacks were acquired at 0.5 $\mu$m intervals for 20 $\mu$m above and below the plane of sharp focus. These stacks of images were used by the PSF-based deconvolution function of the software to calculate and apply the PSF to the experimental stacks of images for deconvolution. The deconvolved stacks were then used to create noise-free 3D reconstructions to determine the relationship of the tumor cells to their surrounding structures. The excitation light source was a 120 watt metal halide lamp (X-Cite120, EXFO Photonics Solutions, Mississauga, ON) with 5% relative output above 600 nm. The camera's CCD is characterized by linear decrease in quantum efficiency from 70% at 450–600 nm to 50% at 700 nm, to 30% at 800 nm, and to 20% at 850 nm.

The PTIR dye-labeled 4T1-GFP cells were either infused directly into the perfusate of the isolated lungs or injected intravenously 3 and 5 days before lung isolation and imaging. Most of the posterior and lateral aspects of the left lung were scanned for the deepest-lying cells that could be focused on in the PTIR dye channels, and the same cells were then imaged in the GFP fluorescence channel. The left lung was chosen because, in the rat, it consists of a single lobe, and thus provided a large surface area that allowed unobstructed scanning for cells.

### 2.4. Statistical analysis

Data analysis was done by SigmaStat (SPSS Inc.) using one way analysis of variance and Bonferroni's test. Data are expressed as means ±SE. Differences were considered significant with $P < 0.05$.

### 3. RESULTS

The mesothelial monolayer of the visceral pleura is transparent with UV and visible light excitation/emission. PTIR dye-labeled 4T1-GFP cells could be readily visualized in the capillaries and pre- and postcapillary vessels of the lungs using both the GFP filter set and the appropriate PTIR dye filter set (Table 1). Lung tissue did not exhibit detectable autofluorescence in the filter windows used to collect emission from the PTIR dyes (S/N= 16 ± 0.4; 17.3 ± 0.6; 16.8 ± 0.5; and

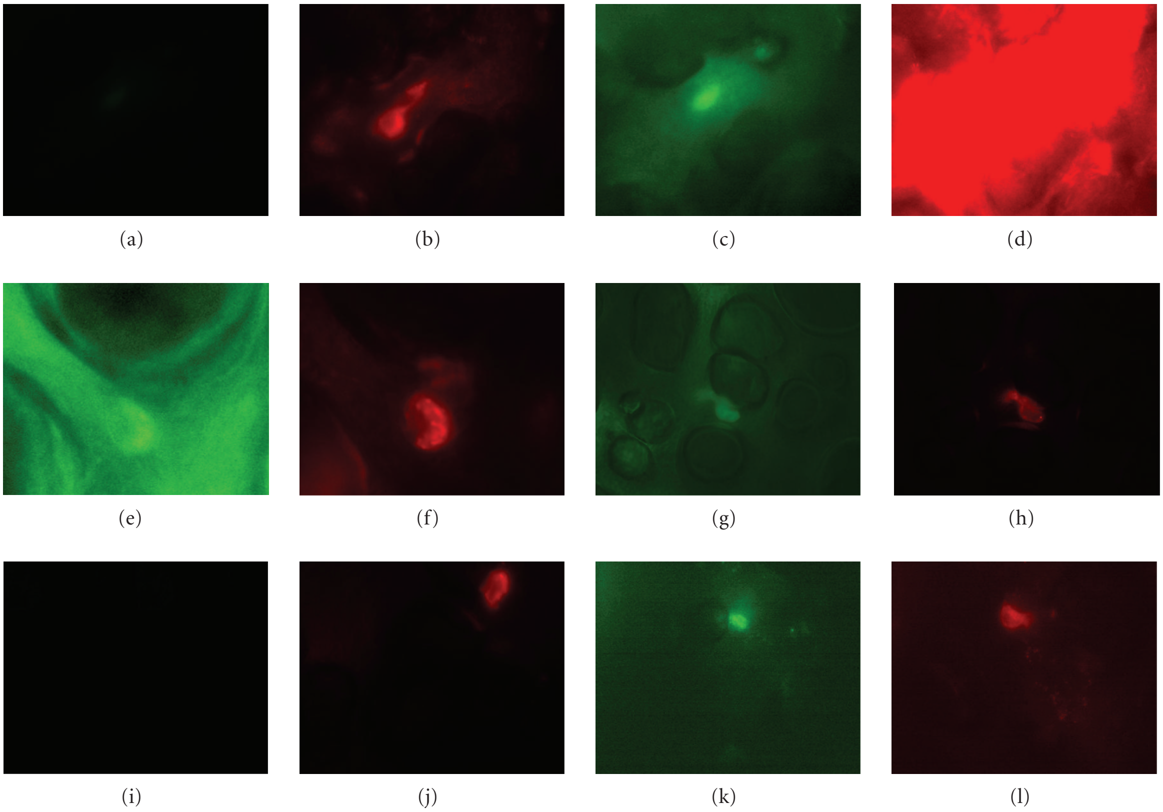

Figure 2: Increased depth of imaging by using far-red and near-IR probes compared with GFP. 4T1-GFP cells labeled with PTIR271–274 probes were administered in the perfusate of isolated rat lungs or administered in the tail vein followed by lung isolation and intact lung transpleural imaging. (a) represents GFP picture (Ex/Em 480/510; exposure 0.5 s; depth from lung surface 35 $\mu$m; (b) represents the same area with PTIR271 picture (Ex/Em 640/680; exposure 0.5 s). (c) and (d) represent same area as in (a) and (b), respectively, but with exposure time of 2 seconds to show the presence of cells in the GFP channel. (e) represents intravascular localization of tumor cell in the GFP channel 5 days after intravenous injection. The vascular outline is visible due to autofluorescence. Exposure time is 2 seconds. Imaging depth is 30 $\mu$m. (f) represents PTIR271 channel picture of the same area as in (e); exposure time is 0.5 second. (g) and (h) represent pictures in the GFP and PTIR272 channels, respectively. Exposure time is 2 seconds for GFP; 0.5 second for PTIR272. Imaging depth is 30 $\mu$m. (i) and (j) represent pictures in the GFP and PTIR273 channels, respectively. Exposure time is 2 seconds for GFP; 0.5 second for PTIR273. Imaging depth is 35 $\mu$m. (k) and (l) represent pictures in the GFP and PTIR274 channels, respectively. Exposure time is 2 seconds for GFP; 0.5 second for PTIR274. Imaging depth is 33 $\mu$m.

17.5 ± 0.9 for PTIR271, PTIR272, PTIR273, and PTIR274, respectively versus 12.5 ± 0.6 for GFP; P < 0.05 for all PTIR probes; $n = 4$). Decreased light scattering was seen with the PTIR dyes than with GFP, as expected due to their longer wavelengths, and was evidenced by increased sharpness and improved edge detection in the PTIR images of tumor cells (Figure 2). The visual quality of pictures was considered optimum when the S/N was higher than 10. The depth at which the cell pictures had S/N of more than 10 was defined as the optimum imaging depth. The average optimum imaging depth for all of the PTIR dyes was higher ($27.8 \pm 0.7\,\mu$m) than for GFP ($20 \pm 0.5\,\mu$m; $p = 0.008$; $n = 50$). The maximum depth at which the cells were still visible (but with S/N < 10) was significantly more for the PTIR dyes ($36.4 \pm 1\,\mu$m) compared with GFP ($30.1 \pm 0.5\,\mu$m; $p = 0.01$; $n = 50$). (Figure 3) shows the values of S/N ratios and imaging depths separately for individual PTIR probes.

Figure 2 shows representative pictures of PTIR dye-labeled 4T1-GFP cells lodged in subpleural lung vessels. As can be readily seen, the long-wavelength probes improve the sharpness and visibility of cells at depths greater than 30 $\mu$m. The PTIR dyes not only improved the quality and depth of images of cells administered acutely in the isolated lung in vitro, but they also survived the challenges in vivo for up to 5 days of study (Figures 2(e) and 2(f)). Although the signal intensity of the PTIR dyes decreased by up to 50% after 5 days in vivo, presumably due to cell division, many tumor cells were still found to generate higher-quality images in the PTIR wavelengths than in the GFP wavelength.

### 4. DISCUSSION

Visualizing biological processes in the living tissue or cells in situ has always been the goal of fluorescence imaging. The collection of such information depends on the optimum

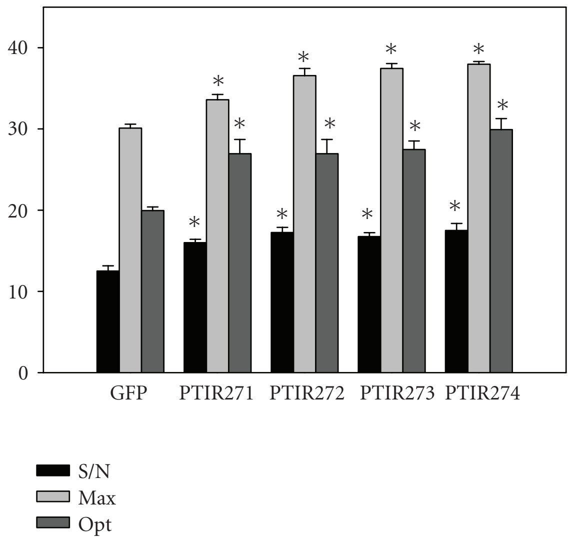

Figure 3: Signal-to-noise ratio (S/N), maximum imaging depth (Max), and optimum imaging depth (Opt) for GFP and long-wavelength PTIR dyes. Maximum imaging depth is the distance between the lung surface and the tumor cell in the lung vessels that still can be imaged using maximum illumination. At optimum imaging depth, high-quality pictures can be obtained with S/N greater than 10. *$P < 0.05$ for all PTIR probes versus corresponding parameters for GFP.

combination of light sources, optics, detection system, image processing, analysis and acquisition hardware and software along with the proper fluorescence probes. The ability to reconstruct the living dynamism in 3D and over time by *z*-axis and time-lapse microscopy has yielded many important insights into the cell biological processes. An important approach more recently taken is the fluorescence imaging of thick living specimens: explant tissues, cells in situ in isolated organs, and whole organisms (e.g., translucent nude mice with GFP-expressing tumor colonies) [14–22]. The major problem encountered in thick tissue imaging is the significant loss of signal at depth due to scattering. Both the excitation light and the emitted light can be absorbed and scattered by the elements of the tissue, thus reducing image quality and limiting the depth of visualization. The maximum depth of imaging allowed by a ×60objective (Nikon PlanApo with a numerical aperture of 1.4 and a working distance of 210 $\mu$m) using a standard 1(1/2) coverslip (170 $\mu$m thickness) is 40 $\mu$m into the tissue. GFP and other visible short-wavelength excitation/emission limit the usable depth of imaging to 20 $\mu$m due to significant scattering and interference caused by autofluorescence.

Using long-wavelength PTIR dyes for imaging intravascular tumor cells in the rat lung instead of GFP increased the effective depth for high-quality imaging from 20 to 28 $\mu$m. This corresponds to a 40% increase in the volume of tissue accessible for imaging. The increase in maximum imaging depth from 30 to 36 $\mu$m results in 20% more sampling volume. The most significant increase in the depth of optimum imaging was associated with the PTIR271 dye as compared with GFP. Shifting to longer wavelengths (PTIR272–274) beyond that led to only minor improvements in the imaging parameters in our system, as expected based on the declining spectral sensitivity of the camera and decreasing light output by the lamp at wavelengths above 700 nm. The data presented herein are not designed for comparative assessment of the efficiencies of the probes because of nonuniform detector sensitivity and illumination. However, our results clearly show that these dyes are suitable for imaging at their far-red and near-IR wavelengths using conventional instrumentation and appropriate filters. Using these new probes in addition to GFP, it should be possible to obtain high-quality images of up to five distinct colors in a single study, a capability that will contribute greatly to the goal of being able to follow biological processes involved in the interactions of multiple cell types.

Retention of the dyes by the plasma membrane permits cell tracking in vivo for up to 5 days in our study. Although permanent expression of GFP or the like is more suitable for very-long-term in vivo tracking, availability of these new FR/NIR dyes will be helpful in multicolor studies. The principle of dye dilution might help estimate the extent of cell division, something that cannot be done with GFP alone [11, 12]. Furthermore, the better tolerance of FR/NIR excitation by cells and fluorescent probes due to lower photon energy and decreased phototoxicity should permit long-term time-lapse imaging.

### 5. CONCLUSION

Use of FR/NIR fluorescent probes offers significant advantages over blue-green fluorophores for live cell imaging in intact organs. Increased depth of imaging, possibility of multicolor imaging, and lesser phototoxicity promise better assessment of different cellular structure and function by fluorescence microscopy.

### 6. ACKNOWLEDGMENTS

We would like to thank Mr. Arthur Groy of PTIR for his assistance in preparing these compounds. Work on this project was supported by NIH Grants R43 CA86692 and R44 EB 00228.

### REFERENCES

[1] Al-Mehdi AB, Tozawa K, Fisher AB, Shientag L, Lee A, Muschel RJ. Intravascular origin of metastasis from the proliferation of endothelium-attached tumor cells: a new model for metastasis. *Nature Medicine.* 2000;6(1):100–102.

[2] Kim JW, Wong CW, Goldsmith JD, et al. Rapid apoptosis in the pulmonary vasculature distinguishes non-metastatic from metastatic melanoma cells. *Cancer Letters.* 2004;213(2):203–212.

[3] Wang H, Fu W, Im JH, et al. Tumor cell $\alpha3\beta1$ integrin and vascular laminin-5 mediate pulmonary arrest and metastasis. *The Journal of Cell Biology.* 2004;164(6):935–941.

[4] Rice BW, Cable MD, Nelson MB. In vivo imaging of light-emitting probes. *Journal of Biomedical Optics.* 2001;6(4):432–440.

[5] Mohlenhoff B, Romeo M, Diem M, Wood BR. Mie-type scattering and non-Beer-Lambert absorption behavior of human cells in infrared microspectroscopy. *Biophysical Journal.* 2005;88(5):3635–3640.

[6] Wilson JD, Foster TH. Mie theory interpretations of light scattering from intact cells. *Optics Letters.* 2005;30(18):2442–2444.

[7] Wunder A, Tung CH, Muller-Ladner U, Weissleder R, Mahmood U. In vivo imaging of protease activity in arthritis: a novel approach for monitoring treatment response. *Arthritis & Rheumatism.* 2004;50(8):2459–2465.

[8] Weissleder R, Tung CH, Mahmood U, Bogdanov Jr. A. In vivo imaging of tumors with protease-activated near-infrared fluorescent probes. *Nature Biotechnology.* 1999;17(4):375–378.

[9] Ntziachristos V, Bremer C, Weissleder R. Fluorescence imaging with near-infrared light: new technological advances that enable in vivo molecular imaging. *European Radiology.* 2003;13(1):195–208.

[10] Morgan NY, English S, Chen W, et al. Real time in vivo non-invasive optical imaging using near-infrared fluorescent quantum dots(1). *Academic Radiology.* 2005;12(3):313–323.

[11] Bercovici N, Givan AL, Waugh MG, et al. Multiparameter precursor analysis of T-cell responses to antigen. *Journal of Immunological Methods.* 2003;276(1-2):5–17.

[12] Poon RY, Ohlsson-Wilhelm BM, Bagwell CB, Muirhead KA. Use of PKH membrane intercalating dyes to monitor cell trafficking and function. In: Diamond RA, DeMagio S, eds. *In Living Color: Flow Cytometry and Cell Sorting Protocols.* New York, NY, USA: Springer; 2000:302–352.

[13] Al-Mehdi AB, Zhao G, Dodia C, et al. Endothelial NADPH oxidase as the source of oxidants in lungs exposed to ischemia or high K+. *Circulation Research.* 1998;83(7):730–737.

[14] Konig K, Schenke-Layland K, Riemann I, Stock UA. Multiphoton autofluorescence imaging of intratissue elastic fibers. *Biomaterials.* 2005;26(5):495–500.

[15] Chen Y, Intes X, Chance B. Development of high-sensitivity near-infrared fluorescence imaging device for early cancer detection. *Biomedical Instrumentation & Technology.* 2005;39(1):75–85.

[16] Citrin D, Scott T, Sproull M, Menard C, Tofilon PJ, Camphausen K. In vivo tumor imaging using a near-infrared-labeled endostatin molecule. *International Journal of Radiation Oncology, Biology, Physics.* 2004;58(2):536–541.

[17] Chen X, Conti PS, Moats RA. In vivo near-infrared fluorescence imaging of integrin $\alpha v\beta3$ in brain tumor xenografts. *Cancer Research.* 2004;64(21):8009–8014.

[18] Hansch A, Frey O, Sauner D, et al. In vivo imaging of experimental arthritis with near-infrared fluorescence. *Arthritis & Rheumatism.* 2004;50(3):961–967.

[19] Gill EM, Palmer GM, Ramanujam N. Steady-state fluorescence imaging of neoplasia. *Methods in Enzymology.* 2003; 361:452–481.

[20] Petrovsky A, Schellenberger E, Josephson L, Weissleder R, Bogdanov Jr. A. Near-infrared fluorescent imaging of tumor apoptosis. *Cancer Research.* 2003;63(8):1936–1942.

[21] Frangioni JV. In vivo near-infrared fluorescence imaging. *Current Opinion in Chemical Biology.* 2003;7(5):626–634.

[22] Eidsath A, Chernomordik V, Gandjbakhche A, Smith P, Russo A. Three-dimensional localization of fluorescent masses deeply embedded in tissue. *Physics in Medicine and Biology.* 2002;47(22):4079–4092.

---

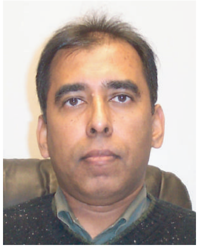

**Abu-Bakr Al-Mehdi** is an Assistant Professor of pharmacology at the University of South Alabama, College of Medicine. Dr. Al-Mehdi received his M.D. (with honors) and Ph.D. degrees in medicine from the Crimea Medical University in Simferopol, Ukraine. He carried out postdoctoral research at the University of Pennsylvania in Philadelphia in vascular and free-radical biology focusing on the mechanisms of endothelial injury with ischemia. Dr. Al-Mehdi postulated the mechanotransduction theory of endothelial response to ischemia, developed an intact organ endothelial cell fluorescence imaging technique, and proposed the intravascular model of hematogenous metastatic tumor growth. His current research interests include role of disturbed flow in mitochondrial function, free-radical mechanisms of atherogenesis, and tumor cell- endothelial cell interactions in tumor neoangiogenesis.

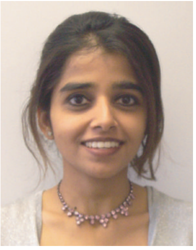

**Mita Patel** received her B.S. degree in biology and chemistry from the University of South Alabama in Mobile, Alabama. After graduation, she worked in the Department of Physiology where she assisted in research on protein kinase C and on cystic fibrosis and mucociliary transport. She is currently doing research in the laboratory of Dr. Al-Mehdi in the Department of Pharmacology. She has become proficient in various laboratory techniques, including fluorescence imaging, isolated lung perfusion, and adapting endothelial cells to different flow conditions.

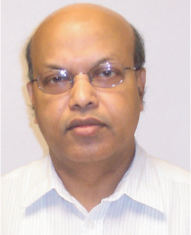

**Abu Haroon** obtained his M.D. and Ph.D. degrees in medicine from the Crimea Medical University in Ukraine. He worked as an Assistant Professor of surgery in the J.I. Medical College, Bangladesh, and as the Chairman of the Department of General Surgery at the City Dental College, Dhaka, Bangladesh. Currently, Dr. Haroon is a Postdoctoral Fellow at the University Of South Alabama, College of Medicne. He has acquired expertise in isolated lung perfusion, experimental and spontaneous metastasis models, and in intact organ fluorescence imaging. His research interests include tumor cell-endothelial cell interactions, mechanisms of pulmonary metastasis, and effects of hypoxia/hyperoxia on the metastatic process.

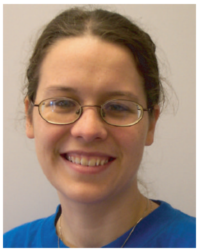

**Darla Reed** obtained her B.S. degree in biology, chemistry, and environmental science from Newberry College in South Carolina. She then entered the Basic Medical Science Graduate Program at the University of South Alabama and is currently pursuing her Ph.D. degree under the mentorship of Dr. Al-Mehdi. She has become proficient in various laboratory techniques, including live-cell fluorescence imaging, isolated lung perfusion, gene transfection, experimental metastasis models, and Fourier analysis of $Ca2^{+}$ oscillations. She has presented her work at the American Association for Cancer Research and Gulf Coast Physiological Society meetings. Her thesis work focuses on the role of mitochondrial $Ca2^{+}$-independent phospholipase A2 in reactive oxygen species production and tumor cell apoptosis.

**B. Ohlsson-Wilhelm** after receiving an A.B. degree in biochemical sciences and a Ph.D. degree in bacteriology from Harvard University, Dr. Ohlsson-Wilhelm carried out postdoctoral research in biophysics at the University of Chicago and in Somatic Cell Genetics at the Institute for Cancer Research (now the Fox Chase Cancer Center). Over a 20-year career in academic medicine, she held senior faculty positions in microbiology, immunology, genetics, and medicine (endocrinology) at the University of Rochester, School of Medicine and Dentistry, and the Pennsylvania State University, College of Medicine at Hershey. Dr. Ohlsson-Wilhelm was subsequently recruited by Zynaxis, Inc., a startup company developing site-selective delivery systems where she rose to Senior Vice President of R&D, with responsibility for programs related to development of improved antineoplastics, antirheumatics, and infectious disease vaccines. In 1996, she and Dr. Katharine Muirhead cofounded SciGro, a biomedical consultancy that works with inventors, entrepreneurs, and investors to translate scientific discoveries and early-stage technologies into successful research tools and products for the pharmaceutical, biotechnology, medical diagnostics, and life sciences industries. Her research interests include cell-based assays and therapies; applications involving flow and/or image cytometry; and drugs and vaccines to treat cancer, immune-based diseases, infectious diseases, and neurological diseases.

**K. Muirhead** earned a B.S degree in chemistry from the University of Wisconsin/Madison, M.S. and Ph.D. degrees in inorganic chemistry from the University of Illinois at Urbana-Champaign, and did postdoctoral research in biochemistry, tumor biology, and analytical cytology. After 6 years on the faculty of the Department of Pathology at the University of Rochester, School of Medicine, she entered the pharmaceutical industry, where she has been involved in discovery research, technology development, and business development for 23 years. At SmithKline Beckman, she served as Senior Investigator in Immunology and Director of the Flow Cytometry Core Facility in R&D. At Zynaxis, Inc., cofounded with colleagues from SmithKline, she rose to Vice President of Research and subsequently Senior Vice President of New Business & Technology Development. In 1996 she and Dr. Ohlsson-Wilhelm cofounded SciGro, a biomedical consultancy that works with inventors, entrepreneurs, and investors to translate scientific discoveries and early-stage technologies into successful research tools and products for the pharmaceutical, biotechnology, medical diagnostics, and life sciences industries. Dr. Muirhead is also a Clinical Assistant Professor at Thomas Jefferson University in Philadelphia. Her research interests include cell tracking; cell-based immunotherapies for cancer and autoimmune diseases; and novel methods for assessment of cell trafficking and function.

**Brian D. Gray** earned a B.S. degree in chemistry in 1980 and a Ph.D. degree in organic chemistry in 1983, both from the University of Dundee, Scotland. His postdoctoral research involved synthesis of various complex organic molecules at Oregon State University and subsequently at SmithKline Beckman in the Department of Macromolecular Chemistry. In 1988, he helped to cofound Zynaxis, Inc. with colleagues from SmithKline, and rose to the position of Director of Medicinal Chemistry. In 1997, he was recruited as Vice President of Chemical Research for PTI Research, Inc. (www.ptiresearch.com), a firm that provides contract and custom chemistry services for life science and pharmaceutical researchers in academia and industry, specializing in the synthesis of heterocyclic organics, bioconjugates, and other compounds requiring complex multistep syntheses. Dr Gray has over 22 years of experience in the design and synthesis of novel compounds for biomedical research, diagnostics, and drug delivery applications, with special expertise in the chemistry of fluorescent dyes and lipophilic agents. His research interests include design and development of novel reagents for neuronal tract tracing (NeuroVue dyes) and cell tracking (CellVue dyes); discovery and development of cell-based diagnostic and drug delivery technologies; and design and synthesis of beads containing ligands for high-throughput screening of ligand-receptor disruptors.

